# Supplementary figures and images for: High-resolution, non-invasive animal tracking and reconstruction of local environment in aquatic ecosystems
Source: Mov Ecol. 2020 Jun 23;8:27. doi: 10.1186/s40462-020-00214-w (PMC7310323; doi:10.1186/s40462-020-00214-w)

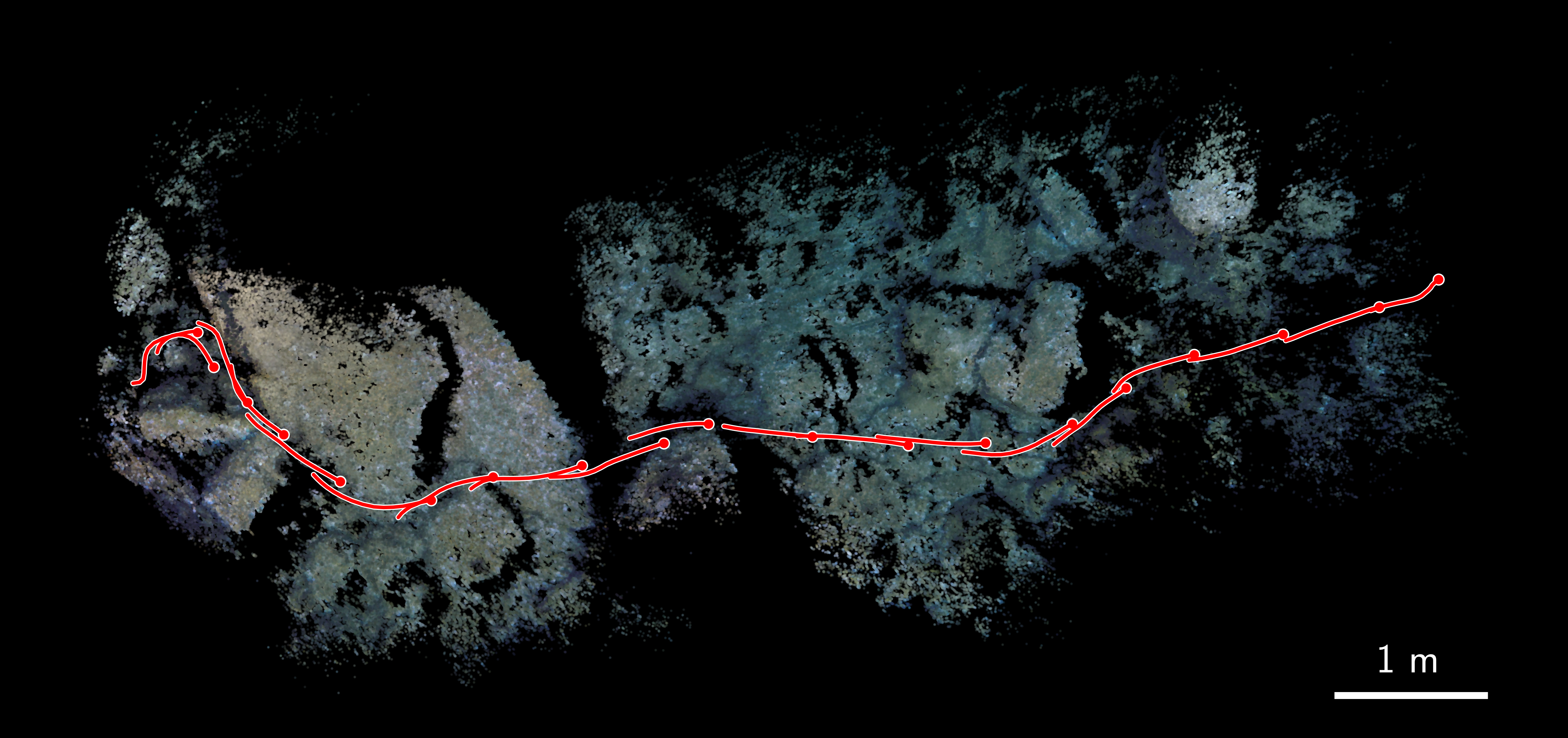

Supplement: Supplementary file 1 — Additional file 1 Tracking results of ’single’ dataset. Top down view of ’single’ results: dense COLMAP 3D reconstruction and trajectories of the tracked animal, C. conger (red). [file 40462_2020_214_MOESM1_ESM.png]

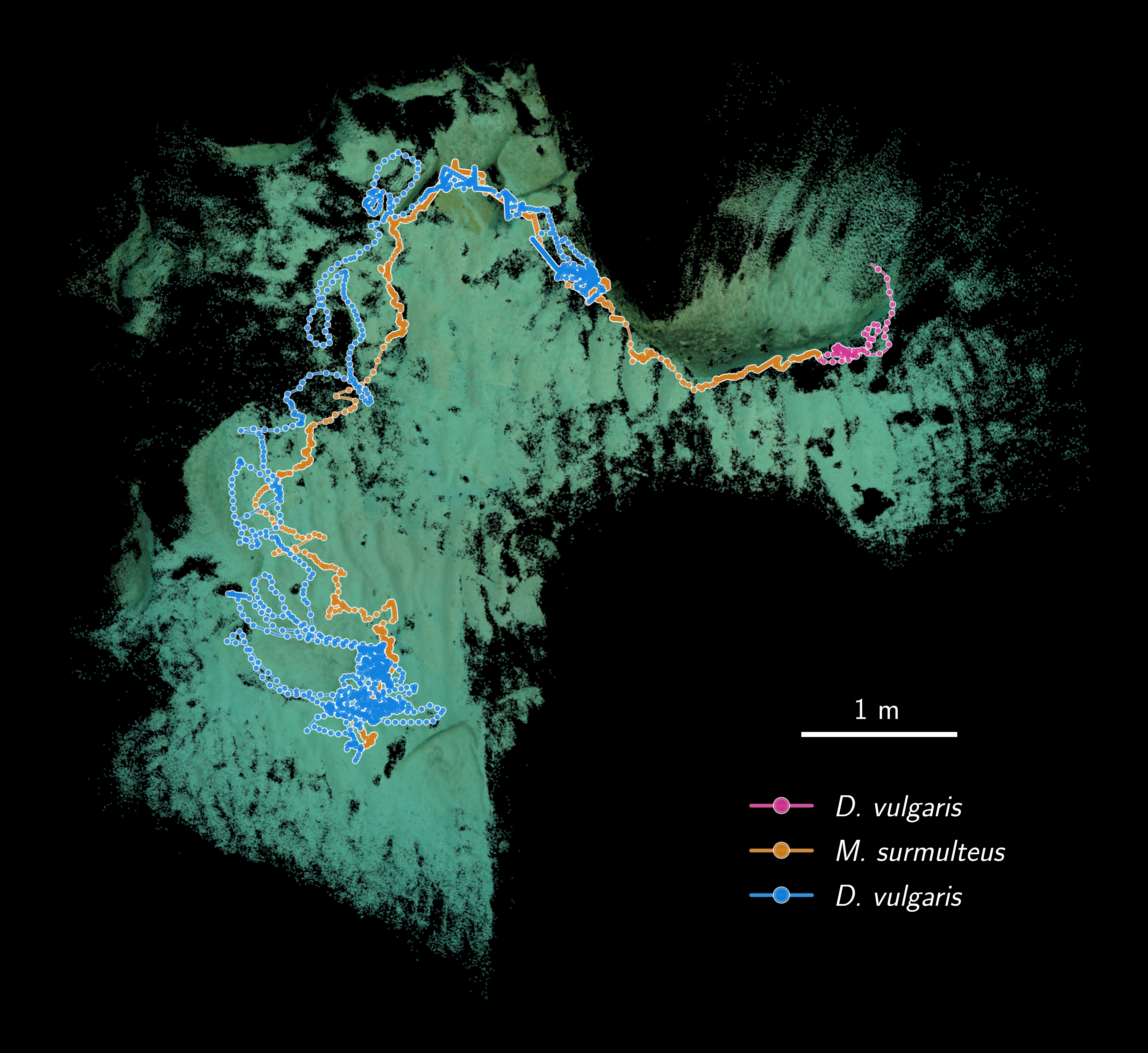

Supplement: Supplementary file 2 — Additional file 2 Tracking results of ’mixed’ dataset. Top down view of ’mixed’ results: dense COLMAP 3D reconstruction and trajectories of the tracked animals, M. surmuletus (orange) and D. vulgaris (purple/blue). [file 40462_2020_214_MOESM2_ESM.png]

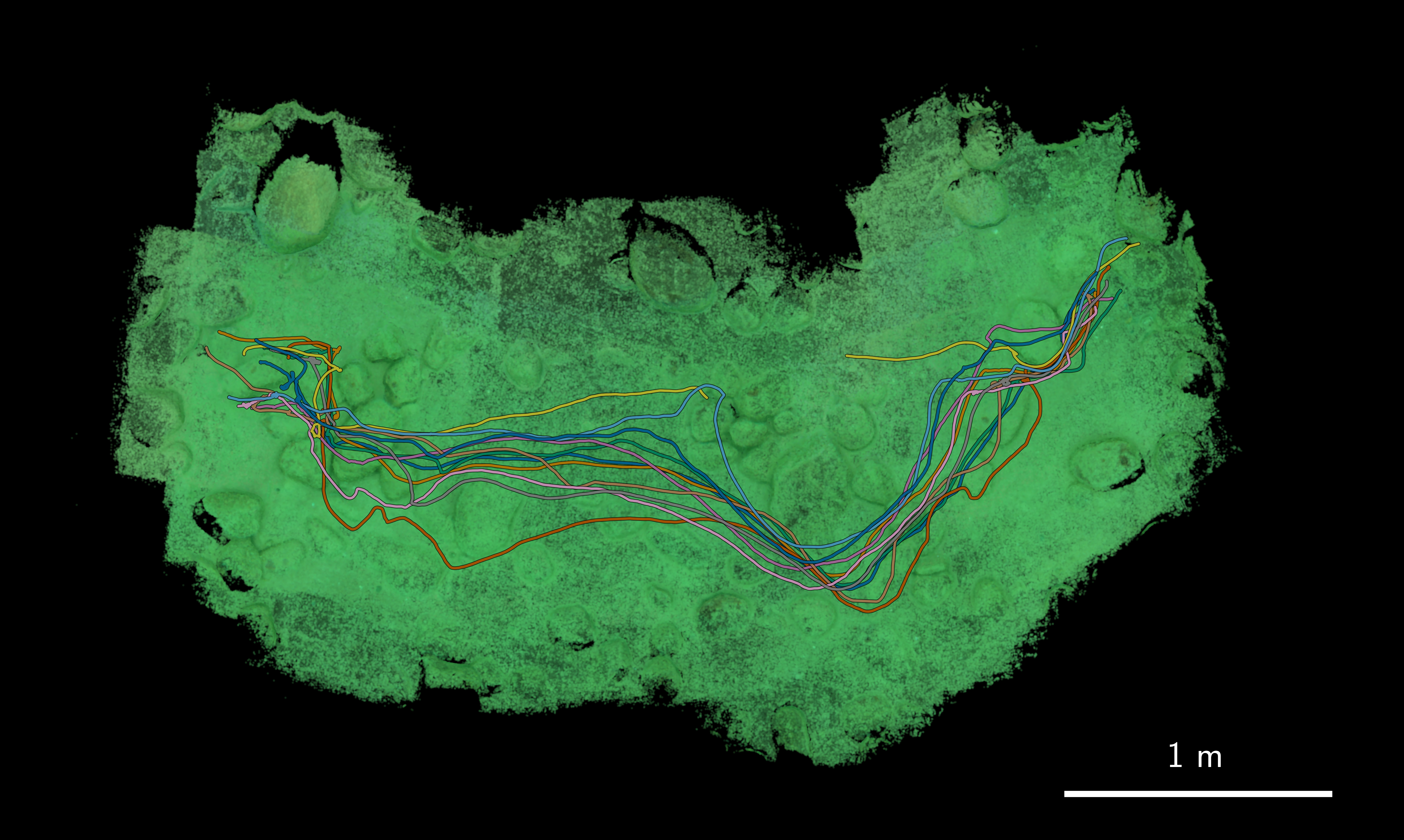

Supplement: Supplementary file 3 — Additional file 3 Tracking results of ’school’ dataset. Top down view of ’school’ results: dense COLMAP 3D reconstruction and trajectories of the tracked animals, L. callipterus. [file 40462_2020_214_MOESM3_ESM.png]
